# Lipid-Based Catalysis Demonstrated by Bilayer-Enabled Ester Hydrolysis

**DOI:** 10.3390/membranes14080168

**Published:** 2024-07-30

**Authors:** Shu Liu, Kiran Kumar, Tracey Bell, Ayyalusamy Ramamoorthy, David Van Winkle, Steven Lenhert

**Affiliations:** 1 Department of Biological Science and Integrative Nanoscience Institute, Florida State University, Tallahassee, FL 32306, USA; sliu6@fsu.edu (S.L.); tbell@bio.fsu.edu (T.B.); 2 Department of Physics, Florida State University, Tallahassee, FL 32306, USA; dvanwinkle@fsu.edu; 3 National High Magnetic Field Laboratory, Florida State University, Tallahassee, FL 32310, USA; kk24a@fsu.edu (K.K.); aramamoorthy@magnet.fsu.edu (A.R.); 4 Department of Chemical and Biomedical Engineering, FAMU-FSU, Tallahassee, FL 32310, USA

**Keywords:** catalysis, partitioning, lipid droplet, vesicle, heterogeneous, biochemistry, pharmacology, synthetic biology

## Abstract

Lipids have not traditionally been considered likely candidates for catalyzing reactions in biological systems. However, there is significant evidence that aggregates of amphiphilic compounds are capable of catalyzing reactions in synthetic organic chemistry. Here, we demonstrate the potential for the hydrophobic region of a lipid bilayer to provide an environment suitable for catalysis by means of a lipid aggregate capable of speeding up a chemical reaction. By bringing organic molecules into the nonpolar or hydrophobic region of a lipid bilayer, reactions can be catalyzed by individual or collections of small, nonpolar, or amphiphilic molecules. We demonstrate this concept by the ester hydrolysis of calcein-AM to produce a fluorescent product, which is a widely used assay for esterase activity in cells. The reaction was first carried out in a two-phase octanol–water system, with the organic phase containing the cationic amphiphiles cetyltrimethylammonium bromide (CTAB) or octadecylamine. The octanol phase was then replaced with phospholipid vesicles in water, where the reaction was also found to be carried out. The reaction was monitored using quantitative fluorescence, which revealed catalytic turnover numbers on a scale of $10^{-7}$ to $10^{-8}$ s^−1^ for each system, which is much slower than enzymatic catalysis. The reaction product was characterized by ^1^H-NMR measurements, which were consistent with ester hydrolysis. The implications of thinking about lipids and lipid aggregates as catalytic entities are discussed in the context of biochemistry, pharmacology, and synthetic biology.

## 1. Introduction

Chemical reactions in biological systems form the basis of metabolism and signaling pathways and are carried out by protein-based catalysts known as enzymes. Enzymes are vital drug targets in treating major diseases due to their role in nearly all physiological and pathological processes [1,2,3]. Globular enzymes are essentially polymeric micelles, and there are also enzymes that are embedded within a lipid bilayer. Enzyme activity is carried out at a catalytic site where the substrate binds and is exposed to specific functional groups such as catalytic triads that directly participate in the chemical reaction [4]. The three-dimensional structure of the enzyme orients these functional groups such that they can coordinate to lower the energy of transition states [5]. One difference between enzymes and traditional synthetic organic catalysts is that enzymes are capable of carrying out molecular recognition, which also depends on the three-dimensional conformation of the enzyme. These processes have come to be understood in terms of an induced fit model [6,7,8,9,10].

Micellar catalysis is a well-established field in organic chemistry, where numerous authors have documented instances of chemical reactions that exhibit accelerated rates in the presence of micelles or vesicles or other organic aggegates [11,12,13,14]. Micelles formed from surfactants in water or other nanoscale compartments are generally thought to provide an environment where organic reactions can take place. Sometimes the micelle forming agents or surfactants are considered catalysts as they speed up the reaction without being consumed, while other times they are thought of more as a solvent or compartment that can solubilize organic compounds to interact with other catalysts in water [14]. Supramolecular catalysis is a field in organic chemistry inspired by enzymes that involves the development of novel synthetic catalysts capable of molecular recognition [15,16]. Within this domain, a key objective involves creating artificial host structures that can effectively control chemical reactivity and catalytic processes. Numerous host structures demonstrate supramolecular catalytic characteristics by developing their recognition abilities [17], particularly when these attributes facilitate the stabilization of intermediate compounds or transition states [18].

The emergence of effective catalysts for biochemical reactions is thought to be a crucial step in the origin and evolution of life [19]. While enzymes are prevalent in today’s cell biology, the discovery of catalytic RNA molecules, known as ribozymes, raised the idea that nucleic acids might have been fundamental to the beginnings of biocatalysts [20,21,22]. In 2001, Lancet et al. introduced the concept of a “lipozyme” as a lipid aggregate that is capable of catalyzing chemical reactions in the context of the lipid world hypothesis for the origin of life [23,24,25,26,27,28]. As the term lipozyme is commonly used to refer to a class of industrial enzymes [29,30], here, we refer to this concept as lipid-aggregate-based catalysis.

While enzymes and synthetic supramolecular catalysts demonstrate molecular recognition through molecular binding events, another way of thinking about this goal is through selective partitioning. For instance, we’ve previously demonstrated partitioning-based molecular recognition in a two-phase octanol/water system [31]. In that model system, the concentration of organic solutes dissolved in the organic phase was found capable of regulating charge-driven selective partitioning of two water-soluble dyes into the organic phase with both kinetic and thermodynamic mechanisms [32]. Similarly, we used a combinatorial materials approach to identify synergistic extraction conditions for the extraction of copper ions from an aqueous phase into an oleic acid organic phase [33]. Phospholipids in bilayer or monolayer form have also been shown to respond selectively to small molecule partitioning, with implications in understanding membrane function [34,35].

Here, we demonstrate the concept of lipid-based catalysis in model systems including an octanol/water two-phase system and in phospholipid vesicles. We demonstrate the possibility of a lipid-bilayer-based catalysis of ester hydrolysis, i.e., esterase activity using an established indicator. Esterases are a ubiquitous class of enzymes that typically hydrolyze esters through a base-catalyzed mechanism [36,37]. Calcein acetoxymethyl ester (Calcein AM) is a widely used indicator of esterase activity in cells [38]. Calcein AM is relatively hydrophobic and not fluorescent but is converted to the anionic fluorophore calcein when taken up by cells and exposed to intracellular esterases [39,40]. We use this compound as an indicator of the esterase activity of a phase-transfer system. This new way of thinking about lipids and lipid aggregates as catalytic entities suggests the importance of considering the local lipid composition of lipid droplets and bilayers.

## 2. Results

**Figure 1:**
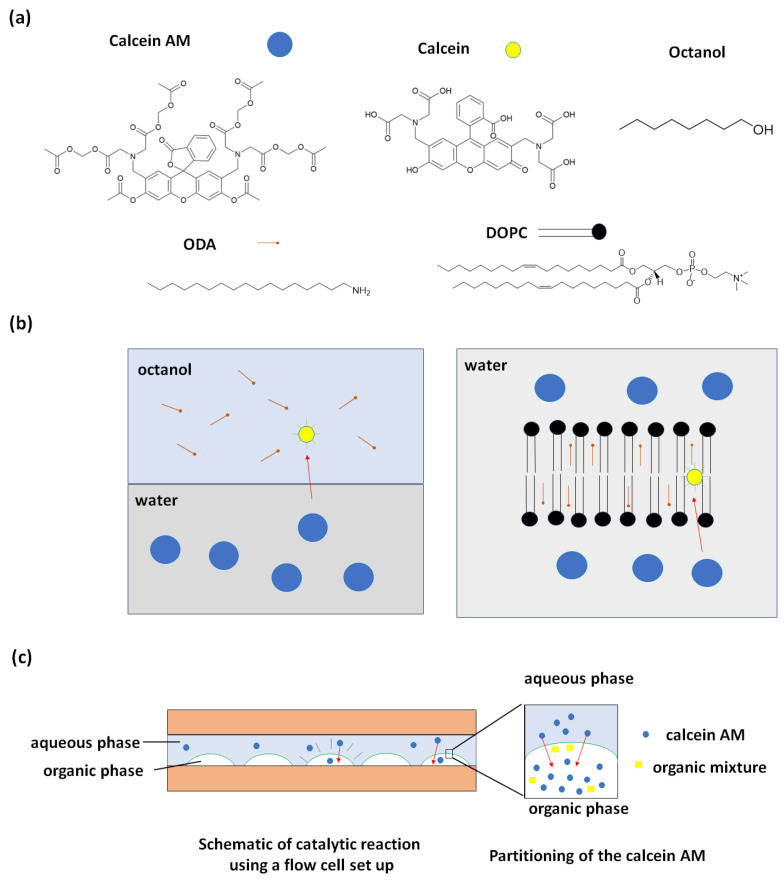
Schematic showing the materials used in this study and the setups to demonstrate the hydrolysis of calcein AM: (**a**) Chemical structure of calcein AM, calcein, octadecylamine (ODA), dioleoylphosphatidylcholine (DOPC), and octanol. (**b**) Schematics of ester hydrolysis of calcein AM in a two-phase octanol–water system (**left**) and in the bilayer of a lipid vesicle (**right**). (**c**) Schematic illustration of a flow cell setup used to observe the reaction.

The chemical structures of the main compounds used here and schematics of the two-phase systems where the reactions are observed are shown in Figure 1. Calcein AM is a colorless hydrophobic dye that contains several ester groups. Calcein is generally expected to be the fluorescent product when the ester groups on calcein AM are cleaved, for instance by intracellular esterases [40]. In particular, the hydrolysis of the two esters on the xanthane backbone and the opening of the lactone ring are necessary for the aromatic conjugation that leads to the fluorescent properties of calcein or any calcein-like product [41]. Octanol was chosen as the organic phase for bulk phase transfer catalysis because it has a polarity similar to that of the hydrophobic region of a lipid bilayer, and for this reason, it is widely used as a standard for characterizing the partitioning of pharmaceuticals [42]. Octadecylamine (ODA) was found to function as a catalyst when included in the organic phase of the two-phase system. The decision to utilize ODA was primarily driven by its primary amine group, which can be expected to both facilitate the partitioning of the anionic product into the nonpolar phase and participate in a base-catalyzed reaction analogous to the histidine residues in active sites of esterases [37]. DOPC (1,2-dioleoyl-sn-glycero-3-phosphocholine) vesicles were used to replace the octanol component as a nonpolar hydrophobic environment suitable for catalysis.

Figure 1b-left shows the schematic of reactions in the octanol–water two-phase system. In this case, we dissolved ODA in octanol and calcein AM in an aqueous solution. The calcein AM molecules transfer from the aqueous phase to the organic or hydrophobic phase where they react in the presence of ODA, resulting in the formation of a fluorescent product such as calcein via ester hydrolysis. Figure 1b-right shows a schematic of the reaction in the presence of a lipid bilayer. ODA was added to the DOPC prior to vesicle formation. When the calcein AM was added to water, it was observed to enter the lamellar lipid bilayer phase and react only in the presence of ODA to generate the fluorescent product.

**Figure 2:**
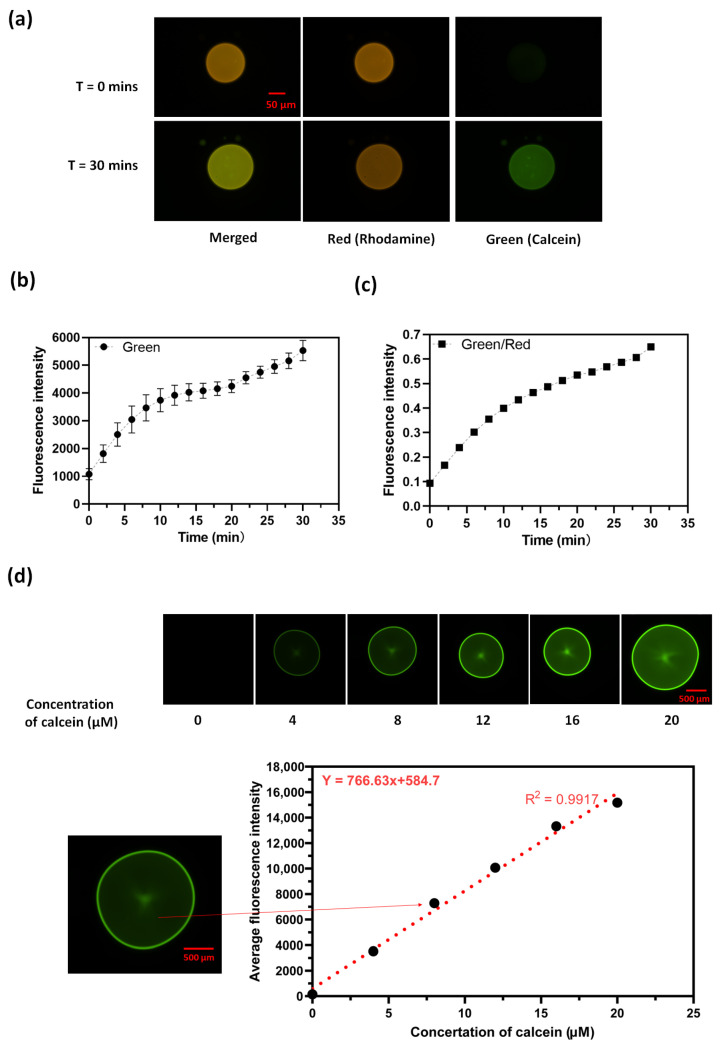
Octanol droplets containing ODA become fluorescent when exposed to aqueous calcein AM: (**a**) Images of ODA/octanol droplets at t = 0 min and t = 60 min under red and green filters and merged channels. (**b**) A plot of average green intensity along with time within the droplets. (**c**) A plot of average green over red along with time within the droplet. (**d**) Calibration of calcein fluorescence in water detected on a fluorescence microscope. **Top**: images of different concertation of aqueous calcein. **Bottom**: a plot of average fluorescence intensity of 16 pixels in the center of the droplet.

To observe the hydrolysis reaction, we first apply a flow cell setup as illustrated in Figure 1c. We spot the lipid droplets on the glass slide, then add a spacer and a second glass slide to form a flow cell. The aqueous 10 µM calcein AM in PBS buffer solution was then flowed into the cell chamber. Figure 2 shows the hydrolysis of calcein AM results using 60 mM ODA in octanol doped with 1 mol% of the fluorescently labeled phospholipid rhodamine PE (1,2-dioleoyl-sn-glycero-3-phosphoethanolamine-N-(lissamine rhodamine B sulfonyl) (ammonium salt)) for visualization of the organic phase. From Figure 2a, we notice that the green intensity increases after 60 min whereas the red intensity as an internal standard doesn’t change. This is evidence that the hydrolysis of calcein AM happens in the presence of ODA in octanol droplets. Control droplets of octanol without ODA did not show catalysis. Figure 2b,c shows the average green and green over red intensity along with time within the octanol droplet. Synthetic calcein was found to partition into octanol drops containing ODA (Figure S1).

To quantitatively determine the concentration of the product generated after the hydrolysis of calcein AM, we calibrated the fluorescence using calcein. This calibration was then used to calculate the catalytic turnover, which refers to the number of substrate molecules that a catalyst converts into a product within a specific time. This value is determined by the maximum velocity of the enzyme-catalyzed reaction, which occurs when the enzyme is completely saturated with substrate. We perform a calibration curve to measure the fluorescence intensity of different concentrations of calcein in the aqueous solution as shown in Figure 2d. Then we calculate the catalytic turnover from the maximum reaction rate which is measured as the slope of the change in fluorescence intensity in the first 2 min of the reaction $v_{t=2\min}=370$ (I/min). Based on the calibration curve in Figure 2d, we convert this value to a reaction rate in units of change in concentration per time $v_{t=2\min}=0.48\;\left(\frac{\mu M}{\min}\right)$ for 60 mM ODA. Finally, the catalytic turnover for ODA equals the max velocity divided by the concentration of the catalyst. If we consider ODA the catalyst, this gives us 1.33 × 10^−7^ $s^{-1}$. This value is comparable to the turnover rate measured by fluorescence in some synthetic systems [43], but it is significantly slower than enzymatic catalysis [44,45,46,47].

**Figure 3:**
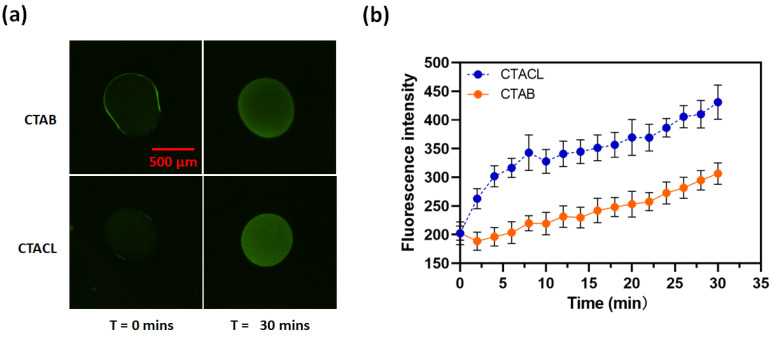
Octanol droplets containing 60 mM CTAB or CTAC become fluorescent when exposed to aqueous calcein AM: (**a**) Images of CTAB and CTAC/octanol droplets at t = 0 min and t = 60 min under green filters. (**b**) A plot of average green intensity along with time within the droplets.

In order to test whether the reaction is catalyzed by the phase transfer agent or is possibly the result of aminolysis, we carried out the reaction using the quaternary ammonium salts cetrimonium bromide (CTAB) and cetrimonium chloride (CTAC) instead of ODA. These quaternary ammonium salts are not capable of participating in aminolysis. Figure 3 shows that octanol drops containing either of these two ammonium salts (60 mM) significantly increase in fluorescence intensity within 30 min, indicating ester hydrolysis of calcein AM. Again, octanol drops that do not contain the amines or ammonium salts do not show any color change. Consequently, it is reasonable to conclude that lipid combinations are capable of catalyzing the ester hydrolysis reaction and the reaction is not the result of aminolysis. However, from this experiment, we cannot exclude the possibility that the CTAB or CTAC might transfer another catalyst from the PBS-buffered aqueous phase into the organic phase.

**Figure 4:**
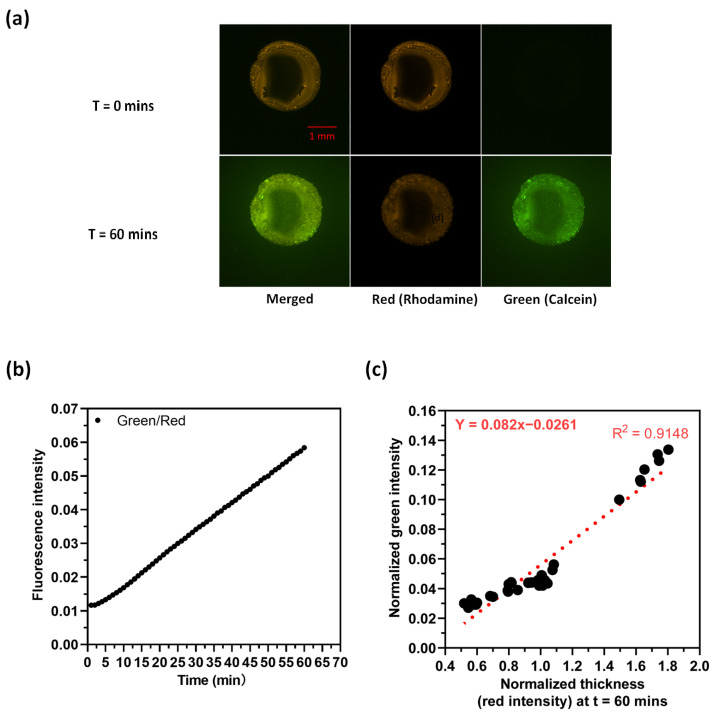
Phospholipid multilayers containing octadecylamine fluoresce when exposed to aqueous calcein AM: (**a**) Images of ODA/DOPC droplets at t = 0 min and t = 60 min under red and green filters and merged channels. (**b**) A plot of average green/red intensity along with time within the droplets. (**c**) A plot of fluorescence intensity with thickness within the droplet.

Figure 4 shows the hydrolysis of calcein AM in surface-supported lipid multilayer films composed of DOPC with 10 mol% ODA. We also included 1% Rhodamine PE into the DOPC as an internal standard. The lipid formulations were mixed in chloroform and spotted onto a glass slide, the chloroform was evaporated in a vacuum, and then a flow cell was constructed by adding a top glass slide. An aqueous solution of calcein AM (10 µM) was then added to the flow cell and the lipid multilayers were monitored by fluorescence microscopy. In Figure 4a, the intensity measured through the red filter remains relatively unchanged between t = 0 min and t = 60 min. However, the intensity measured through the green filter shows an increase in the same time frame. Figure 4b clearly indicates that the intensity ratio of green/red rises over time, and the green intensity increases as shown in Figure S2, indicating hydrolysis of the calcein AM. Phospholipid multilayers without ODA did not show any increase in fluorescence. We used the red intensity as a measure of droplet thickness [48] and plotted its relationship with the green intensity normalized to the thickness at t = 60 min, as depicted in Figure 4c. This plot reveals a linear correlation between droplet thickness and green intensity, with a slope of 0.082, closely aligning with the 0.06 value shown in Figure 4b. It’s worth noting that our analysis is based on 40-pixel values within the droplets, leading to a minor variance between the slope and the actual green/red intensity at t = 60 min due to the limited data points. This indicates that the phospholipid DOPC is a suitable replacement for the octanol for this phase transfer catalysis reaction, with the fluorescent product partitioning into the phospholipid multilayer volume.

**Figure 5:**
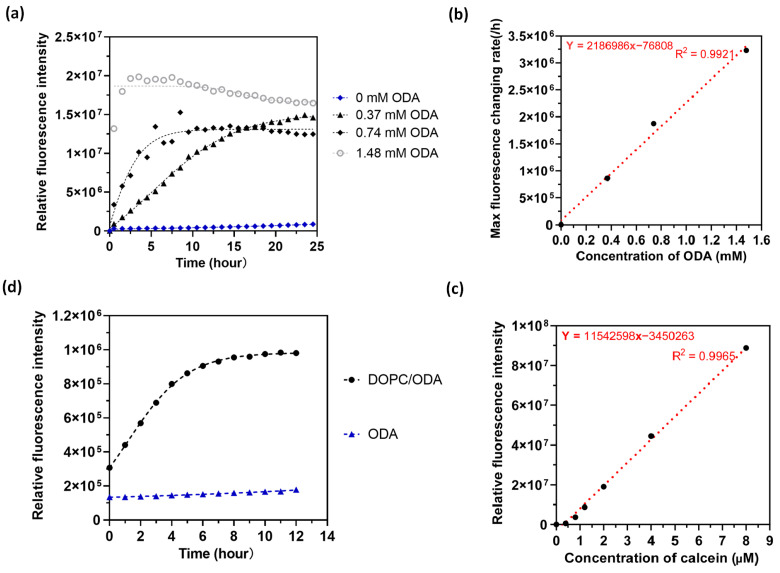
Lipid vesicle solution-based ester hydrolysis: (**a**) Fluorescence intensity along with time for different concentrations of octadecylamine in DOPC vesicles. (**b**) Calculated max fluorescence changing rate with different concentrations of octadecylamine to obtain the catalytic turnover number with linear fitting. (**c**) Calibration of a plate reader with different concentrations of calcein in 96 well plates. (**d**) Fluorescence intensity along with time for ODA vesicles solution with/without DOPC.

It is apparent that the fluorescence product partitions into the lipid multilayer, and in order to quantify the catalytic turnover in the lipid-based system we would like to distinguish partitioning from the catalysis. We therefore carried out the experiment in vesicles using a bulk solution and measured fluorescence intensity in a plate reader. Multilamellar vesicle solutions in water were prepared containing 0, 0.37, 0.74, and 1.48 mM of ODA and 3.2 mM DOPC in water. Calcein AM solution was then added to the vesicle solution to obtain a final concentration of 10 µM and fluorescence was measured over time. Figure 5 shows the result of the kinetic of fluorescence intensity measured by a plate reader. From the data, we notice that the initial rate of change in fluorescence increases as the concentration of ODA increases. So, we calculated the maximum reaction rate by using linear fit for the first five data points. Figure 5b shows the relationship of ODA concentration with the maximum reaction rate. From Figure 5b, we noticed that the maximum reaction rate increases with the concentration of ODA in the linear region, and the equilibrium intensities for different concentrations of ODA reaching the same level, which demonstrates that the addition of ODA can catalyze the hydrolysis of calcein AM in a synergistic way in the presence of vesicles [49]. We fit the curve in the linear region and obtain the catalytic turnover rate in Figure 5b based on the calibration curve in Figure 5c, and the catalytic turnover rate equals 5 × 10 ^−8^ s^−1^, which again is comparable to the turnover rate measured by fluorescence in some synthetic systems [43] but significantly slower than enzymatic catalysis [44,45,46,47]. However, it is likely the rate could be increased by optimizing the conditions of the catalysis, including the composition of the lipid-based catalyst in more heterogeneous systems. Figure 5d displays the fluorescence results for ODA vesicles with and without DOPC. The data reveal that the fluorescence intensity remains constant over time when DOPC is absent, indicating that the combination of ODA and DOPC in the vesicles is crucial for catalyzing the hydrolysis of calcein AM.

**Figure 6:**
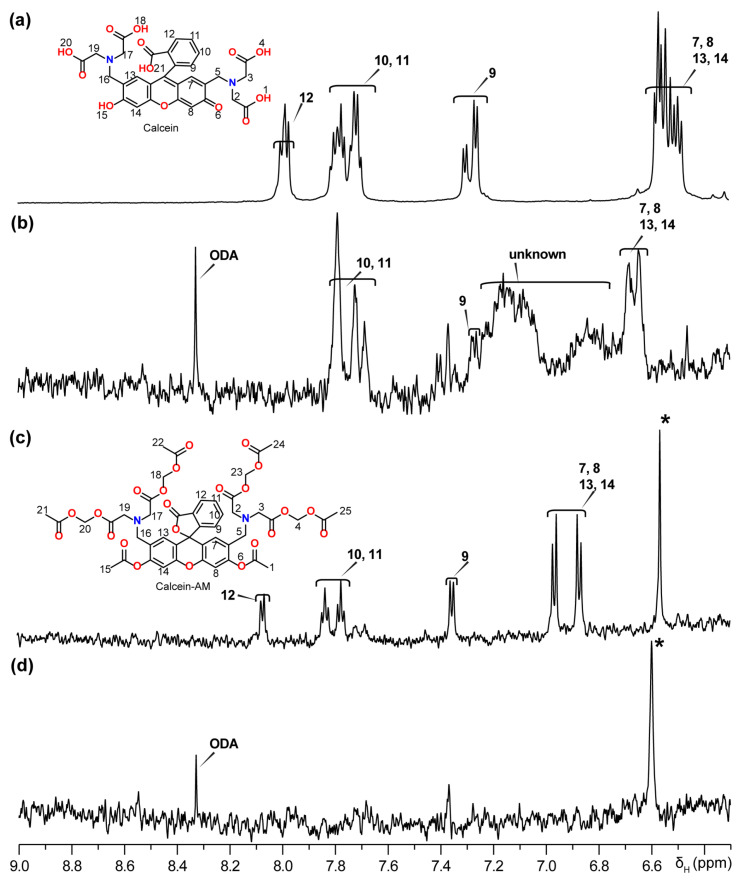
^1^H NMR spectra and assignments of calcein molecules: (**a**) 500 µM calcein-A, (**b**) 100 µM calcein AM incubated with 3.2 mM DOPC and 1.85 mM ODA, (**c**) 100 µM calcein AM, and (**d**) 3.2 mM DOPC and 1.85 mM ODA. All samples were dissolved in deuterated DMSO-d6 solvent, and experiments were performed at 25 °C using a 600 MHz NMR spectrometer (full spectra are provided in Figure S3). Assigned protons are numbered and * denotes impurities.

^1^H-NMR experiments were used to further characterize the ester hydrolysis reaction. ^1^H NMR spectra are shown in Figure S3. Since the concentration of lipids used was much higher than calcein AM, we focused our analysis only on the aromatic region that is devoid of any peaks from lipids. Figure 6 shows the aromatic regions of ^1^H-NMR spectra of synthetic calcein as a positive control (Figure 6a), the reaction product after incubating calcein AM in 3.2 mM DOPC/1.85 mM ODA vesicles (Figure 6b), pure calcein AM as a negative control (Figure 6c), and 3.2 mM DOPC/1.85 ODA vesicles without calcein AM (Figure 6d). Table S1 summarizes the chemical shift values measured for the assignment of aromatic peaks based on reported NMR spectra for eosin Y, a similar molecule [50], and 2D COSY and TOCSY NMR assignment (Figures S4 and S5). In order to detect signals from calcein AM and hydrolyzed compounds, higher concentrations (100–500 µM) and volumes (600 µL) of the calcein AM solutions were needed in the NMR samples than that used in previous experiments. Due to the high cost of calcein AM, we had to use a lower concentration (100 µM), which resulted in lower signal-to-noise ratio spectra for solutions requiring this compound. Interestingly, the DOPC + ODA with calcein AM sample exhibited ^1^H NMR peaks corresponding to both the reactant (i.e., calcein AM) and product (i.e., similar to calcein) in addition to peaks from DOPC and ODA. Peaks 7, 8, 13, and 14 appear in different locations in calcein and calcein AM, which is understandable due to the different aromatic nature of the xanthene core. In the reaction product, we also identified peaks 9, 10, and 11 corresponding to both calcein and calcein AM with significant overlap. It is noticeable that peak 12 does not appear with the same chemical shift in the reaction product and a precise assignment of peak 12 was not possible in the reaction product due to peak shift and peak overlap. This shift or overlap may be explained as the calcein produced from the reaction, likely in equilibrium with lactone ring close–open structures, or possibly due to the ODA containing a positively charged amino group which could serve as a counter-ion to neutralize the carboxylic acid group [41]. The observed broad ^1^H peak(s) in the aromatic region of the DOPC + ODA and calcein AM sample is likely due to the presence of more than one type of “calcein-like” molecules. Although the mechanism of the reaction remains elusive, based on the ^1^H-NMR results and fluorescence data, we conclude that the reaction product contains a product with a calcein-like aromatic region (Figure 6b). The fluorescence generated by the product can be used as an indicator of hydrolysis.

We aimed to assess whether the amino group alone drives the catalysis of calcein AM hydrolysis. To this end, we conducted experiments using solutions of calcein AM with liquid ethylamine, as both ethylamine and octadecylamine possess the same amino group. Figure S6 presents the results from the plate reader for 10 µM calcein AM with 66% liquid ethylamine, and pure calcein AM as a control. It was observed that the calcein AM with ethylamine did not catalyze the reaction. This lack of activity might be attributed to ethylamine being in liquid form at room temperature, unlike solid octadecylamine. This, along with the observation that ODA without an organic phase or lipid bilayer also does not catalyze the reaction indicates that a nonpolar environment is necessary for the reaction to occur.

## 3. Discussion

We observed the hydrolysis of calcein AM in aqueous form within an octanol–water phase system, as well as in lipid multilayer and vesicle solutions in the presence of the cationic amphiphiles octadecylamine or CTAB. The catalytic turnover numbers determined by quantitative fluorescence measurements were found to be on a scale of $10^{-7}$ to $10^{-8}$ s^−1^ for each system which significantly slower than enzymatic catalysis. This is likely due to the need for millimolar concentrations of the amine or ammonium salts to observe the reaction at room temperature. If one considers the catlaytic entity to be an aggregate rather than an individual molecule, turnover would be much higher. Furthermore, it is worth noting that the fluorescence intensities were calibrated in an aqueous solution, while the brightness of the fluorescent product may be sensitive to the nonpolar environment. However, the values obtained were found to be reproducible in both the octanol droplet and the vesicle-based systems. These values therefore suggest that lipid aggregates have the potential to catalyze chemical reactions. The cationic amphiphiles appear to be catalysts for the reactions described here in the traditional sense of organic chemistry. However, as the cationic amphiphiles by themselves proved ineffective in catalyzing the reaction, the presence of a nonpolar or hydrophobic environment also appears crucial to the reaction mechanism.

The idea of organic aggregates speeding up chemical reactions is well-established in synthetic organic chemistry. For example, phase-transfer catalysis is a valuable method used extensively in various chemical fields. This technique facilitates reactions between reagents that are in different phases and might not readily interact due to their separation. By introducing a phase transfer agent, one of the reagents is moved to a position where it can efficiently react with another [51,52,53]. CTAB (cetyltrimethylammonium bromide) [13,54] and ODA (octadecylamine) [55,56] have been pivotal as early micellar and/or phase transfer catalysts in chemical processes [57]. ODA, for instance, has been effectively used to transfer platinum nanoparticles from water to organic solvents by forming a hydrophobic layer around the nanoparticles, enabling their dispersion in nonpolar environments. This technique underscores the significant role of such catalysts in both academic research and industrial applications [58,59]. However, in our system, the amine or ammonium groups are introduced into the organic phase. We can’t exclude the possibility that they may be bringing a counterion such as chlorine, bromide, or other ion from the aqueous phase that may participate in the reaction. However, as synthetic calcein was observed to preferentially partition into the ODA-containing octanol phase, it seems likely that the cationic amphiphiles may stabilize the product in the hydrophobic environment by neutralizing the charge of the carboxylic acids on the hydrolyzed product. The precise mechanism of the reaction described here, however, remains undetermined.

Considering that the number of possible lipid mixtures in a cell scales in a way comparable to nucleic acid or protein sequence information [23,31], it is likely that these rates could be significantly increased through combinatorial formulation [33]. As lipid formulations have been shown to be capable of partitioning-based molecular recognition [32,34,35], lipid composition may play a larger role in catalysis than simple bilayer compartmentalization. This suggests that there may be value in looking for reactions that may take place in hydrophobic environments in vivo, and to consider the role of local lipid composition and heterogeneity in metabolism and signaling [60]. For example, Coenzyme Q [61] and vitamin [62] K are examples of lipids with functional groups that directly participate in well-characterized enzymatic reactions, and are therefore good candidates for the identification of lipid-based catalysts in vivo.

The concept of lipid-based catalysis has several implications for pharmaceuticals. First, if lipid-based catalysis could be identified in vivo, it may serve as an innovative class of drug targets. As the majority of FDA-approved drugs are lipophilic, it is possible that some of them may already function by affecting local lipid composition. Second, as lipids are widely used for drug delivery and formulation [63], the idea that certain formulations may catalyze reactions could be used for therapeutic benefit. For instance, lipid-based catalysts could be designed to mimic the specificity and efficiency of traditional enzymes but in nonaqueous environments. This opens up possibilities for developing new drug delivery systems where the drug release can be controlled catalytically within lipid-based carriers [64].

Finally, as the idea of lipid-based catalysis was conceived in the context of origin-of-life research [23], the possibility of lipid-based catalysis has implications for synthetic biology. Efforts to modify organisms for technological applications or build new life-like systems from a bottom-up approach can benefit from nonpolymeric catalysis. It is still somewhat of a mystery why cells invest so many resources into generating highly diverse lipids, with approximately 5% of existing organisms’ genes being used to generate the lipidome [65]. The presence of lipid-based catalysis could justify this investment. Phase-separated, lipid-based systems such as micelles, vesicles, and droplets of highly heterogeneous composition are promising systems for the emergence of biological phenomena.

## 4. Conclusions

This work demonstrates the capability of a lipid bilayer to provide an environment suitable for lipid-based catalysis. Hydrolysis of the nonfluorescent calcein AM to form the fluorescent calcein product was monitored using fluorescence to track the reaction. The reaction was found to be readily catalyzed by octadecylamine, but only in the presence of a nonpolar environment such as octanol or the hydrophobic region of a lipid bilayer. The kinetics of the reaction were characterized in both the octanol-based and bilayer-based systems, and were found to be on the scale of $10^{-7}$ to $10^{-8}$ s^−1^, which is comparable to some slower enzymes. ^1^H NMR experiments confirm the formation of a calcein-like product. As heterogeneous lipid bilayers are abundant in living organisms, the results have implications for the role of lipids in regulating chemical reactions in biology.

## 5. Methods

### 5.1. Materials

Calcein acetoxymethyl ester (calcein AM) was purchased from VWR (4591070). ODA (L15458) was purchased from Alfa Asear Chemical (Ward Hill, MA, USA). CTAB (H5882) and calcein (154071) were purchased from Millipore-Sigma (St. Louis, MO, USA). DOPC (850375) and Rhodamine PE (810150) were purchased from Avanti polar lipids (Alabaster, AL, USA). 1-Octanol (222920) was purchased from Beantown Chemicals (Hudson, NH, USA). Chloroform (for HPLC, ≥99.9%) was purchased from Sigma-Aldrich (St. Louis, MO, USA) PBS buffer (137 mM NaCl, 2.7 mM KCl, 9.5 mM Phosphate buffer, pH = 6.6–7.2) was diluted to 1× from a 10× solution purchased from VWR (K813-500ML) (650498).

### 5.2. Vesicle Preparation

Multilamellar vesicle solutions were prepared as follows. 1 mM stock solutions of calcein AM were prepared. These solutions were diluted 100× with PBS buffer to result in 10 μM solutions. DOPC and ODA solutions in chloroform were prepared and the chloroform was evaporated in a vacuum at 10 mbar for 5 min. We maintained a fixed quantity of DOPC molecules and adjusted the volume of ODA to alter the molecular ratio between DOPC and ODA for testing the reaction with calcein AM.

### 5.3. Fluorescence Microscopy

Fluorescence microscopy was performed using a Ti-E inverted microscope (Nikon Instruments, Melville, NY, USA) to measure the optical response of the droplets. A 4× objective lens was used for magnification. The exposure time was set to 1 s for both the TRITC filter (excitation at 544 nm, emission at 570 nm) and the FITC filter (excitation at 490 nm, emission at 515 nm) to measure the fluorescence intensity of the droplets. The lamp intensity was set to 16 on the ND scale, corresponding to a transmittance of 6.25%.

### 5.4. Fluorescence Characterization

Fluorescence was measured using a Molecular Devices plate reader integrated with the SoftMax Pro software. The lipid vesicle solution was put in the 96-well plate. We use the kinetics function to measure the absorbance and fluorescence of the lipid vesicles for more than 10 h. We selected a medium gain setting to measure the relative fluorescence intensity of the solutions. If the intensity reached its maximum, we switched to a low gain setting and performed another measurement. The fluorescence function is measured with an excitation wavelength of 480 nm and an emission wavelength of 520 nm. The absorbance measurement was performed using a scanning function of wavelengths from 230 nm to 1000 nm with step 10 nm.

### 5.5. Flow Cell Setup

The droplets of mixtures were spotted onto the substrate. After that, we used polydimethylsiloxane (PDMS) as the spacer and covered the top with a glass slide. The aqueous solution flowed into the cell, and we recorded the optical response of the droplet arrays using the fluorescence microscope.

### 5.6. NMR

DOPC and ODA vesicle solutions were prepared as described previously and exposed to calcein AM. Once the reaction was completed, samples were lyophilized overnight to remove any residual water and were redissolved in deuterated dimethyl sulfoxide (DMSO-d6). Control calcein AM, calcein, and DOPC/ODA samples were directly dissolved in DMSO. NMR experiments were carried out on a 600 MHz NMR spectrometer equipped with a broadband BBI probe. Two-dimensional COSY and TOCSY spectra were recorded using standard pulse programs available in the NMR spectrometer’s library with 16 scans and 128 or 256 complex points. A relaxation delay of 2 s was used throughout 2D experiments. DMSO solvent peak was used as an internal reference for ^1^H chemical shifts. All NMR data were processed with Topspin 4.3.0 software (from Bruker).

## Data Availability

The original contributions presented in this study are included in the article/Supplementary Material; further inquiries can be directed to the corresponding author.

## Supplementary Materials

The following supporting information can be downloaded at https://www.mdpi.com/article/10.3390/membranes14080168/s1, Table S1: 1H chemical shift values measured in DMSO-d6 at 25 °C. Tentative resonance assignment is based on reported NMR spectra for similar molecules and 2D NMR assignment (Figures S4 and S5); Figure S1: Calcein partitions into octadecylamine/octanol drops; Figure S2: Phospholipid multilayers containing octadecylamine fluoresce when exposed to aqueous calcein AM. A plot of average green intensity along with time within the droplets. Figure S3: 1H NMR spectra and assignments of calcein molecules: (a) 500 µM calcein, (b) 100 µM calcein-AM incubated with DOPC and ODA, (c) 100 µM calcein-AM, and (d) DOPC and ODA. All samples were dissolved in deuterated DMSO-d6 solvent and experiments were performed at 25 °C using a 600 MHz NMR spectrometer. * Peaks likely from impurities. Figure S4: 2D 1H-1H COSY spectrum of 500 µM calcein in deuterated DMSO-d6 solvent and experiments were performed at 25 °C using a 600 MHz NMR spectrometer. Figure S5: 2D 1H-1H TOCSY spectrum of 500 µM calcein in deuterated DMSO-d6 solvent and experiments were performed at 25 °C using a 600 MHz NMR spectrometer. Figure S6: Fluorescence measurements were taken using aqueous calcein AM and calcein AM mixed with ethylamine in a plate reader. The results clearly show that calcein AM does not hydrolyze into fluorescent calcein in the presence of ethylamine.

## List of Abbreviations

DOPC	1,2-dioleoyl-sn-glycero-3-phosphocholine
Calcein AM	Calcein acetomethyl ester
ODA	Octadecylamine
CTAB	Cetyltrimethylammonium bromide
CTAC	Cetrimonium chloride
DMSO	Dimethyl sulfoxide
