# Supplementary material for: Lipid-Based Catalysis Demonstrated by Bilayer-Enabled Ester Hydrolysis
Source: Membranes (Basel). 2024 Jul 30;14(8):168. doi: 10.3390/membranes14080168 (PMC11356346; doi:10.3390/membranes14080168)
Supplement: Supplementary file 1 [file membranes-14-00168-s001.zip › membranes-3062484-supplementary.pdf]

Supporting information

Lipid-based catalysis demonstrated by bilayer enabled ester hydrolysis

Sample	δ_H (ppm)				
^{1}H label	7, 8	9	10, 11	12	13, 14
Calcein	6.52	7.28	7.75	7.98	6.52
Calcein-AM+DOPC+ODA	6.68	7.27	7.75	-	6.69
Calcein-AM	6.92	7.35	7.80	8.07	6.92

Table S1. ^{1}H chemical shift values measured in DMSO-d6 at 25 $^{\circ}$C. Tentative resonance assignment is based on reported NMR spectra for similar molecule (Okuom et al, 2013) and 2D NMR assignment (**Figure S4 and S5**).

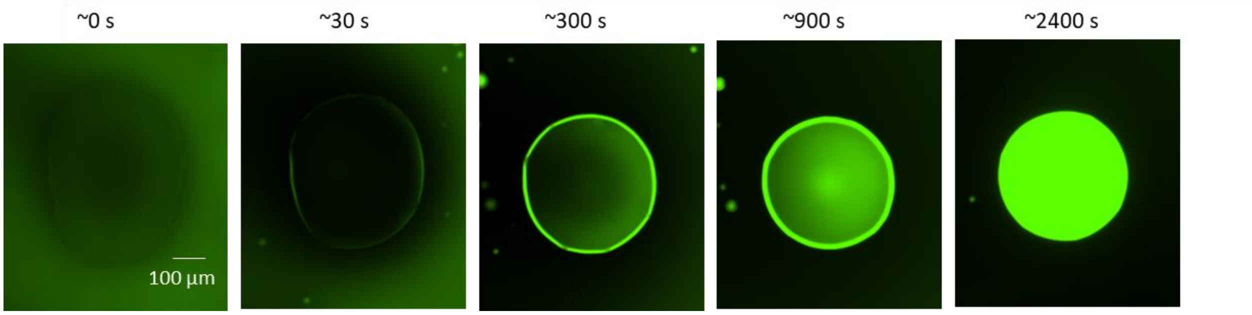

Figure S1. Calcein partitions into octadecylamine/octanol drops.

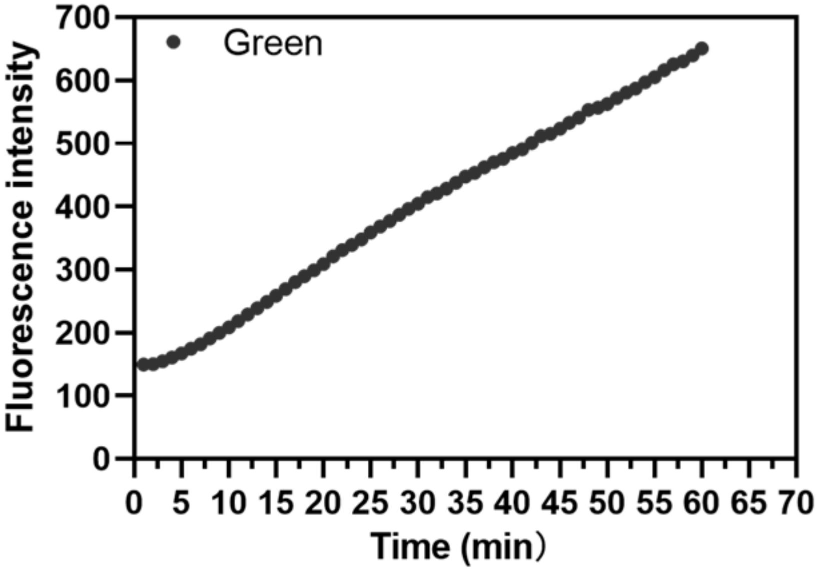

Figure S2. Phospholipid multilayers containing octadecylamine fluoresce when exposed to aqueous calcein AM. A plot of average green intensity along with time within the droplets.

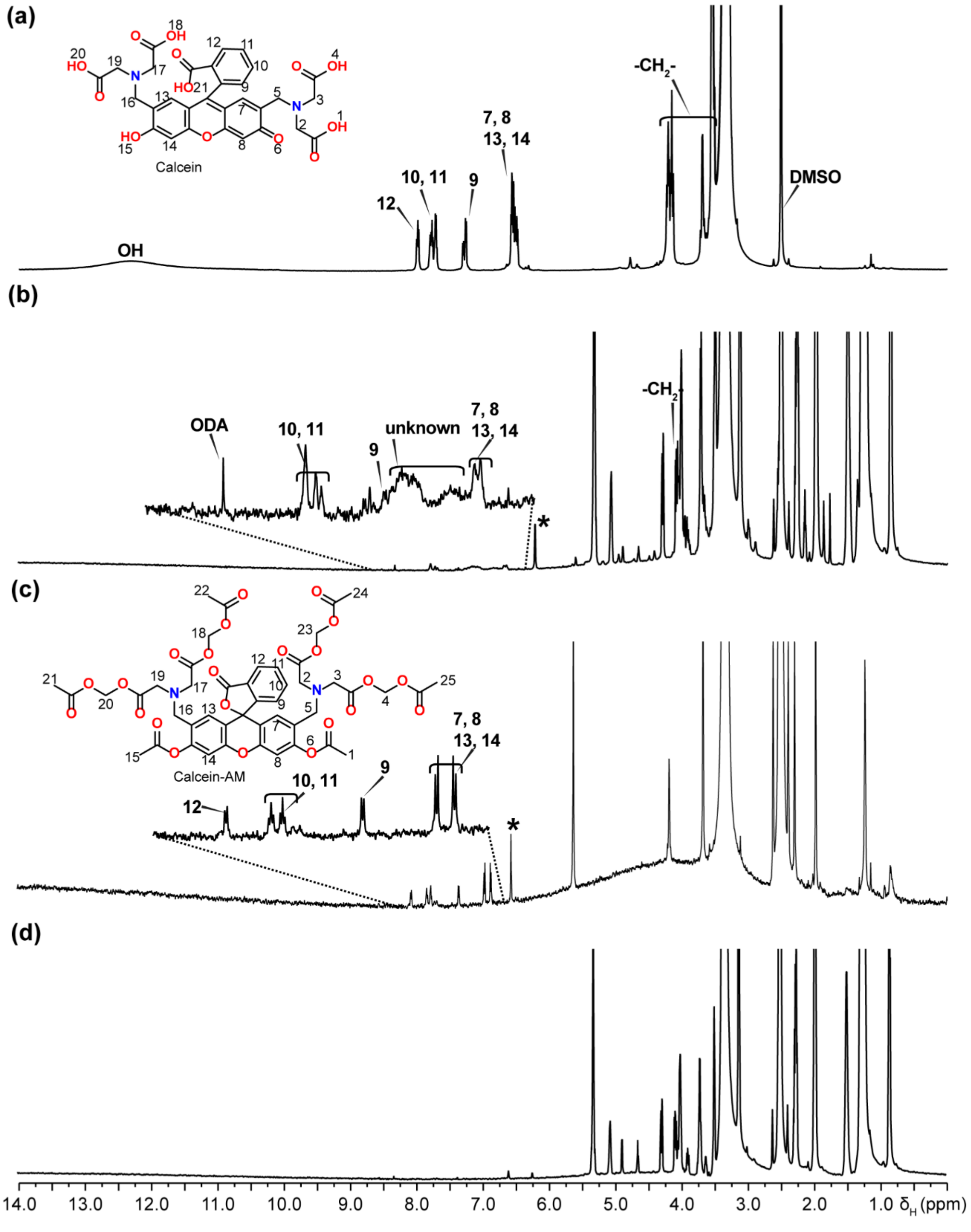

Figure S3. ^{1}H NMR spectra and assignments of calcein molecules: (a) 500 μM calcein, (b) 100 μM calcein-AM incubated with DOPC and ODA, (c) 100 μM calcein-AM and (d) DOPC and ODA. All samples were dissolved in deuterated DMSO-d6 solvent and experiments were performed at 25 °C using a 600 MHz NMR spectrometer. *Peaks likely from impurities.

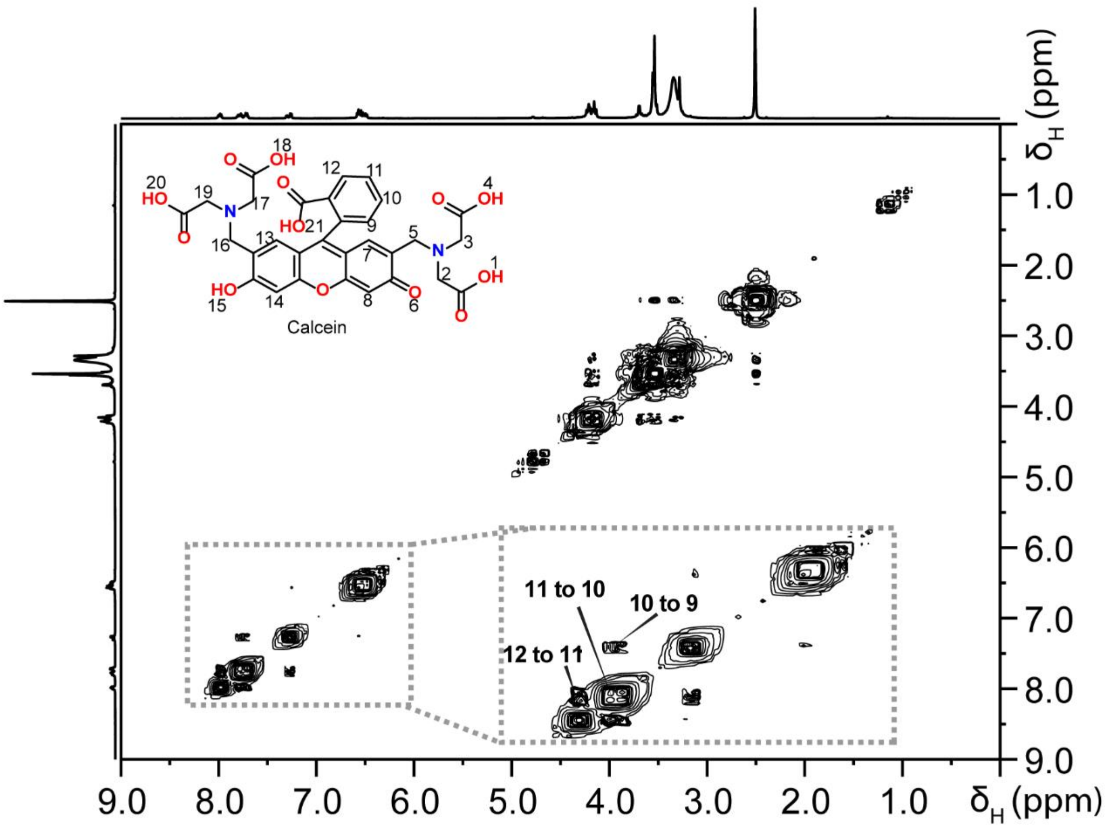

Figure S4. 2D ^1H-^1H COSY spectrum of 500 μM calcein in deuterated DMSO-d6 solvent and experiments were performed at 25 °C using a 600 MHz NMR spectrometer.

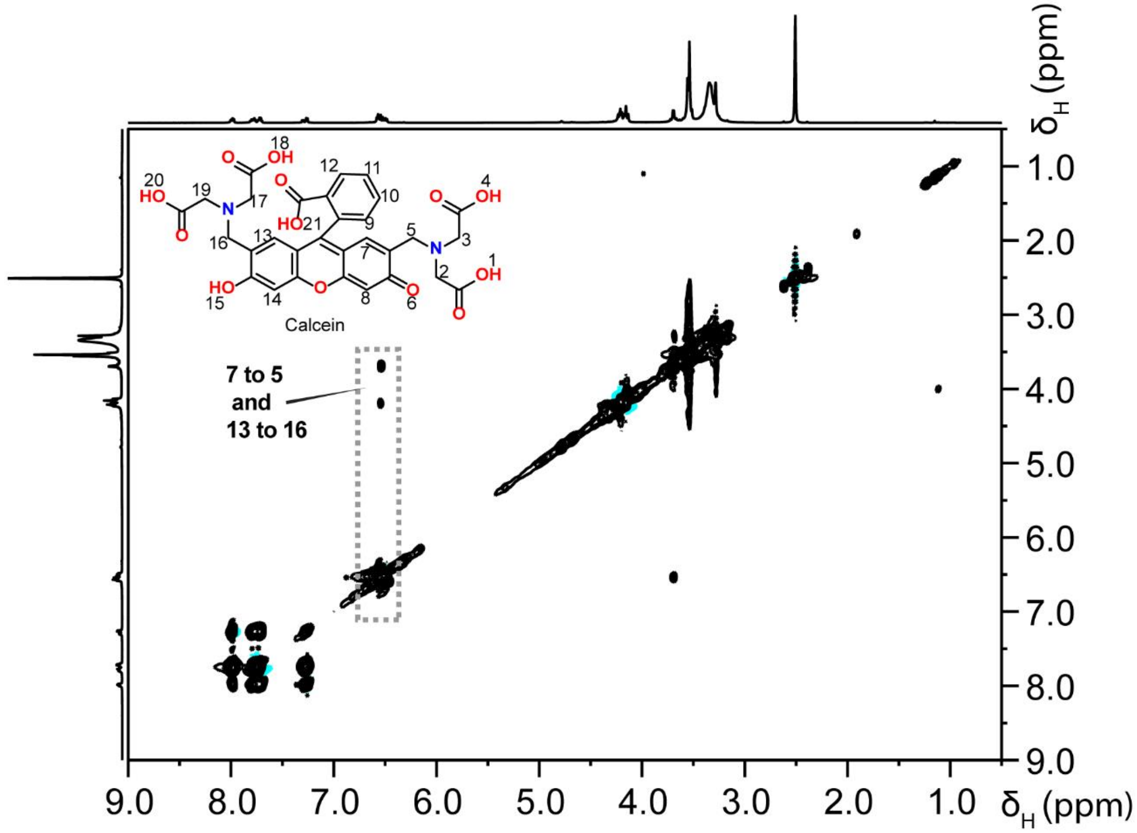

Figure S5. 2D ^{1}H-^{1}H TOCSY spectrum of 500 μM calcein in deuterated DMSO-d6 solvent and experiments were performed at 25 °C using a 600 MHz NMR spectrometer.

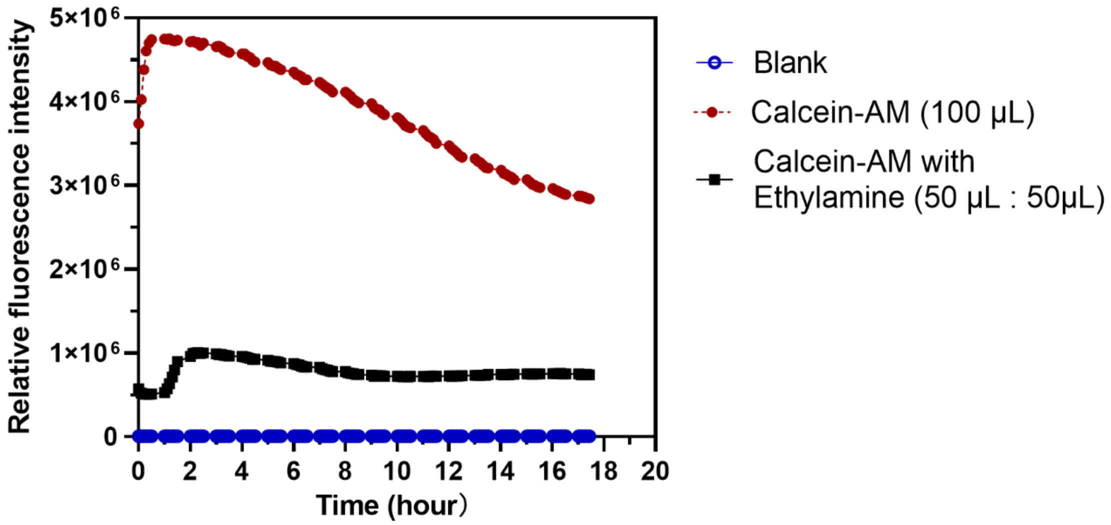

Figure S6. Fluorescence measurements were taken using aqueous calcein AM and calcein AM mixed with ethylamine in a plate reader. The results clearly show that calcein AM does not hydrolyze into fluorescent calcein in the presence of ethylamine.
